# Effect of Supplemental Protease on Growth Performance and Excreta Microbiome of Broiler Chicks

**DOI:** 10.3390/microorganisms8040475

**Published:** 2020-03-27

**Authors:** Jeferson M. Lourenco, S. Claire Nunn, Eliza. J. Lee, C. Robert Dove, Todd R. Callaway, Michael J. Azain

**Affiliations:** 1 Department of Animal and Dairy Science, University of Georgia, Athens, GA 30602, USA; jefao@uga.edu (J.M.L.); eayton88@uga.edu (E.J.L.); crdove@uga.edu (C.R.D.); mazain@uga.edu (M.J.A.); 2 College of Veterinary Medicine, University of Georgia, Athens, GA 30602, USA; cn210@uga.edu

**Keywords:** 16S rRNA, amino acids, chicks, feed efficiency, microbiome, protease, *proteus*

## Abstract

One-day-old chicks were assigned one of four dietary treatments in a 2 × 2 factorial design in which the main effects were diet (adequate vs. low protein) and the addition of protease (0 vs. 200 g/1000 kg of feed). Chick performance (days 0–14) was recorded and their excreta were analyzed for short chain fatty acids, ammonia, and composition of the microbiota using 16S rRNA gene sequencing. Birds fed the low protein diet had lower body weight gain and poorer overall feed conversion ratio (FCR) (*p* ≤ 0.04); however, these parameters were not affected by the inclusion of protease (*p* ≥ 0.27). Protease inclusion did not affect any particular bacterial genus in the excreta, but it increased the total number of observed OTUs (*p* = 0.04) and Faith’s phylogenetic diversity (*p* = 0.05). Abundance of *Proteus* and *Acinetobacter* were lower in the excreta of chicks fed the low protein diet (*p* = 0.01). Abundance of *Bacteroides* was associated with poorer FCR, while *Proteus* was associated with improved FCR (*p* ≤ 0.009). Although diet had a stronger impact than protease on chick performance, both diet and protease yielded some changes in the intestinal microbiotas of the birds.

## 1. Introduction

Feed costs are estimated to contribute between 70% and 80% of the total cost of raising poultry [1]. As a result, there is great interest in feed additives that can improve nutrient availability in feeds. The use of supplemental enzymes added to feed represent one means of improving nutrient availability. Currently, the feed enzyme business has over 1 billion USD in sales annually, with most of this accounted for by phytases, proteases, and carbohydrases [2]. Exogenous enzymes are included in rations to improve production efficiency and growth performance and have been estimated to save the global feed market 3 to 5 billion USD a year [1,3].

Proteases have the potential to improve growth performance in poultry because the chicken’s pancreatic protease activity is low at hatching and increases up to 21 days of age [4]. Due to the immaturity of chicks’ digestive systems, digestive protease may be limiting the protein digestion in young birds. Exogenous proteases may complement pancreatic enzymes and increase the rate of intestinal protein degradation [5]. Besides improving protein digestibility, supplemental proteases may reduce the requirement for amino acids [6].

It has been demonstrated that exogenous enzymes can change gut morphology, pancreatic enzyme production and secretion, the microbial populations along the gastrointestinal tract, and the short chain fatty acid profile in the digesta [7,8,9]. Previous work has shown that the chicken gastrointestinal microbiome plays an important role in intestinal development [10] and can have a significant influence on bird’s health and growth performance [11]. Exogenous proteases could change the extent that feed substrates are degraded and modified in the chicken digestive system, and potentially change the nutrients used by the chicken microbiota and the chicken microbial population itself [12].

Most research on the use of proteases has been in combination with other enzymes, while few studies have been performed on mono-component proteases, and these studies have had variable results [5,13,14,15]. Furthermore, only a few studies have examined the effects of exogenous enzymes on the chicken microbiome using next-generation DNA sequencing (e.g., 16S rRNA gene sequencing) [16,17], and fewer have examined the impact of protease [18,19]. Therefore, the objective of this study was to investigate the effect of exogenous protease on young chicks’ growth performance, as well as the impacts on their excretal microbiota, and concentrations of end products such as short chain fatty acids and ammonia. Our hypothesis was that protease would improve protein digestibility, modify the birds’ intestinal microbiota, and ultimately improve growth performance during the first 14 days of their lives.

## 2. Materials and Methods

### 2.1. Ethics Statement

All procedures involving live animals were verified and approved by the University of Georgia’s Office of Animal Care and Use (Animal Use Protocol #A2017-08-012-Y1-A0). The chicks used in this study were housed at the University of Georgia Large Animal Research Unit, located at the Department of Animal and Dairy Science in Athens, GA.

### 2.2. Animals and Treatments

One-hundred-and-sixty one-day-old, mixed-sex Cobb broiler chicks were obtained from a local commercial hatchery, group-housed in a battery brooder, and fed a nutritionally complete starter diet for 24 h after arrival. Birds were then randomly assigned to 20 battery brooder pens (average initial wt. 48.64 ± 0.75 g). A total of 8 chicks were placed in each pen. The pens were randomly assigned, with 5 pens per dietary treatment. Chickens were fed one of four dietary treatments: (1) a diet with adequate levels of crude protein and essential amino acids (1.31% total lysine); (2) the same adequate-protein diet with the addition of protease (200 g/1000 kg feed); (3) a lower crude protein diet that was marginally deficient in amino acids (1.15% total lysine); (4) the low-protein diet with the addition of protease (200 g/1000 kg feed). Nutrient levels in the adequate protein diet met or exceeded those recommended for the birds [20]. It should be noted that while the National Research Council (NRC) publications on nutrient requirements for each species are usually the standard, the most current NRC for Poultry was published in 1994 [21] and is not accurate for modern lines of broiler chickens [22]. The low protein diet was formulated by reducing lysine level and maintaining a pattern of amino acids relative to lysine similar to that in the adequate diet. All other nutrients, except for essential amino acids, were at or above the requirement. The commercial protease (Vitazyme PRO; Vitech Ultra Bioscience Corporation; Orange, CA, USA) is a product of fermentation of *Aspergillus Niger*. According to the manufacturer, this protease has the ability to release tyrosine from casein. As previously mentioned, this protease was added at 200 g/1000 kg of feed in two of our four treatments. Composition of the basal diets and their nutritional content are presented in Table 1.

All treatments were fed in mash form. Birds had ad libitum access to feed and water throughout the entire study. The room was kept at a constant temperature (29–31 °C) with continuous lighting throughout the 14-day experimental period. Bodyweight and feed intake were measured on days 0, 7, and 14. Additionally, average daily gain (ADG) and feed conversion rate (FCR) were calculated. Samples of excreta were collected on day 14 and stored in a freezer at −20 °C until further analysis. This collection was performed individually on each pen by gathering the excreta accumulated in the base of the pen during the previous 24 h, since they had been cleaned the day before. Upon collection, a sample from each pen was placed in a sterile 50 mL conical tube and was immediately frozen, until further analyses were performed.

### 2.3. Ammonia Analysis

Excreta ammonia concentration was determined using the phenol-hypochlorite reaction described by Weatherburn [23]. Briefly, a 25 µL sample (1:10 excreta to water, vortexed) or standard (0 to 11.99 mM ammonium sulfate) was mixed with 3.0 mL phenol reagent followed by 3.0 mL hypochlorite solution and was incubated at 39 °C for 20 min, and absorbance was read at 630 nm on a Thermo Scientific Genesys 30 Visible Spectrophotometer (Thermo Fisher Scientific, Waltham, MA, USA). Ammonia concentration was calculated from the standard curve and expressed as mg/g of excreta.

### 2.4. Short Chain Fatty Acid Analysis

Chicken excreta were mixed with water at a 1:3 ratio and was then centrifuged at 10,000× *g* for 10 min. Supernatants were combined with 25% *w/v* solution of meta-phosphoric acid at a 5:1 ratio, vortexed, and frozen overnight. After thawing, the samples were again centrifuged at 10,000× *g* for 10 min and the supernatant was mixed with ethyl acetate in a 1:2 ratio. The top layer was transferred to a vial for gas chromatography analysis using a Shimadzu GC-2010 Plus gas chromatograph (Shimadzu Corporation, Kyoto, Japan) equipped with a flame ionization detector and a capillary column (Zebron ZB-FFAP; 30 m × 0.32 mm × 0.25 μm; Phenomenex Inc., Torrance, CA, USA). Sample injection volume was set as 1.0 μL, and helium was used as the carrier gas. Column temperature was initially set at 110 °C and gradually increased to 200 °C. Injector and detector temperatures were set at 250 °C and 350 °C, respectively.

### 2.5. DNA Extraction and Sequencing

DNA was extracted by placing 0.33 g of feces into lysing matrix tubes and processing according to a hybrid DNA extraction protocol [24]. Briefly, this protocol consisted of a combination of a mechanical method using the FastDNA Spin Kit for Feces (MP Biomedicals, Solon, OH, USA) and an enzymatic method based on the QIAamp DNA Stool Mini Kit (QIAGEN, Valencia, CA, USA). The concentration of DNA resulting from each sample was determined spectrophotometrically using the Take3 plate in conjunction with the Synergy H4 multimode plate reader (BioTek, Winooski, VT, USA). Library construction and sequencing were performed by the Georgia Genomics and Bioinformatics Core at the University of Georgia (Athens, GA, USA). PCR libraries were generated using the S-D-Bact-0341-b-S-17 (5′-CCTACGGGNGGCWGCAG-3′) forward and S-D-Bact-0785-a-A-21 (5′-GACTACHVGGGTATCTAATCC-3′) reverse primer pair [25]. Sequencing was performed on an Illumina MiSeq using a v3 600 cycle kit (Illumina, San Diego, CA, USA).

### 2.6. 16.S rRNA Gene Sequencing

Paired-end sequencing reads were analyzed using the software package QIIME v1.9.1 [26]. Sequences were chimera checked against the Greengenes 13_8 database [27] and clustered into operational taxonomic units (OTUs) according to their sequence similarity (97%). Sequences were aligned using PyNAST [28] and a phylogenetic tree was subsequently produced. Singleton OTUs and OTUs whose representative sequences could not be aligned with PyNAST were removed. Alpha and beta diversities and OTU richness were calculated after sample sizes were standardized to 56,855 sequences. Alpha diversity indexes were computed using QIIME’s “alpha_rarefaction.py” script. The computed indexes were: Shannon diversity index, Chao1, Faith’s phylogenetic diversity, Evenness, and number of observed OTUs. Beta diversity between all pairs of samples was calculated using QIIME’s “beta_diversity_through_plots.py” script, which generates a beta diversity matrix with the dissimilarity between every pair of samples and runs the principal coordinate analysis. The results for the unweighted UniFrac distances were visualized using EMPeror [29].

### 2.7. Statistical Analysis

Statistical analyses were performed using the software Minitab 18^®^ and R v2.15.1 (R Foundation for Statistical Computing, Vienna, Austria). Data were analyzed as a 2 × 2 factorial design with diet (adequate protein vs. low protein) and protease (0 vs. 200 g/t) as factors, as well as their interaction. Comparisons across groups were carried out by ANOVA using pen as the experimental unit. Regression analysis was also performed on bacterial genera that were significantly correlated with overall FCR (*Bacteroides* and *Proteus*), and results were plotted. Results were considered significant at *p* ≤ 0.05 and treated as trends when 0.10 ≥ *p* > 0.05.

## 3. Results and Discussion

### 3.1. Growth Performance

In this study, diet did not have a significant impact on daily feed intake (*p* ≥ 0.16; Table 2); however, birds fed the lower crude protein diet had significantly lower final body weight (*p* = 0.03). Moreover, ADG from day 0 to 7, from day 7 to 14, and overall ADG (day 0 to 14) were all greater in chicks fed the adequate protein diet (*p* ≤ 0.04). Similarly, FCR for all the evaluated periods was improved for chicks fed the adequate protein diet (*p* ≤ 0.04). These results concerning bird performance are not surprising since amino acid deficiency is known to reduce growth rate and feed efficiency in broilers. Thus, our results are consistent with the findings from previous studies [13,14,30]. Contrary to what was observed for protein levels in the diet, the presence of protease did not have a significant impact on any of the animal performance traits evaluated, except for FCR assessed in the first week of the study (i.e., days 0 to 7; *p* = 0.03). Furthermore, no significant interaction between the diet and protease was observed (*p* ≥ 0.19) for any of the performance traits. Collectively, our findings do not support our initial hypothesis that protease would improve growth performance in neonatal chicks.

The mechanism, kinetics, and preferred substrates of exogenous proteases are not well understood [6]. Proteases break down proteins by hydrolyzing peptide bonds of specific amino acids. Protease selectivity depends on the accessibility of the peptide bonds within the substrate. For instance, denatured proteins are more easily degradable than compact proteins, which resist enzyme action. Moreover, proteases differ in their source (most commercial proteases are isolated from bacteria or fungi), optimal pH, mode of action, and preferred substrate [3,6,31]. Measuring growth performance and nutrient digestibility are common ways to evaluate commercial enzymes; however, these approaches provide little information on how these specific enzymes actually function [3]. Consequently, many specific questions on how commercial proteases function within the chicken’s gastrointestinal tract remain unanswered.

In previous studies, supplemental protease in the diet from 1 to 14 days had no effect on body weight gain, feed intake, and feed efficiency of chickens fed soybean-meal diets [32,33]. Cowieson et al. also failed to observe, on days 7–14, a protease effect on bird body weight gain and feed intake, but protease appeared to increase the gain to feed ratio [34]. Interestingly, in all of these studies, a positive effect of protease on body weight gain and feed efficiency was observed after 14 days [32,33,34]. This indicates that exogenous protease efficacy can be impacted by the age of the birds. It has been shown that age affects the secretion of endogenous trypsin. Noy and Sklan observed that the release of endogenous trypsin into the duodenum is not very efficient until day 21 of age [35]. In addition to age, diet is known to influence pancreatic output and enzyme composition [6]. Although more research is needed, there is evidence that the addition of exogenous protease to the diet reduces pancreatic production and secretion of endogenous proteolytic enzymes [1,15,32,36,37,38], an effect that could even result in a decrease in protein digestibility [38]. Although pancreatic enzyme secretion was not quantified in the present study, a similar physiological response may have occurred in our birds, resulting in the lack of protease impact on chick performance.

### 3.2. Microbial Fermentation Byproducts: Ammonia and Short Chain Fatty Acids

Ammonia is a marker of microbial fermentation and a byproduct of the deamination of amino acids [39]. As shown in Table 3, the adequate protein diet without protease had numerically greater (*p* = 0.13) excreta ammonia concentration than the low protein diet. Lowering dietary crude protein tends to decrease excreta ammonia concentration [39]; however, in the present study, the differences in crude protein and amino acids between the adequate and low protein diets might not have been extreme enough to result in differences in the excreta ammonia concentration. Additionally, no protease effect or interaction effect between diet and protease was observed on the concentration of excreta ammonia (*p* ≥ 0.17). In their meta-analysis, Lee et al. concluded that if birds are performing well, a response due to protease addition is unlikely [40]. Therefore, the lack of response to protease on excreta ammonia concentration may be due to the fact that both diets in our study provided enough amino acids to meet the birds’ requirements for growth.

Short chain fatty acids are end products of bacterial fermentation of carbohydrates and amino acids. Table 3 shows that, among all treatments, acetate was found at the greatest concentration (87–89%), followed by butyrate (6–8%), propionate (0.9–2%), and small amounts of the others, which were as expected at the chickens’ age [9,10]. The majority of short chain fatty acids were likely produced from the fermentation of carbohydrates that escaped digestion in the small intestine. Some gastrointestinal microbes ferment amino acids and can deaminate them rapidly [41]. Therefore, the trend (*p* = 0.09) for a greater concentration of short chain fatty acids in the excreta of chicks fed the diet with higher protein level seems logical.

Reduction in dietary crude protein tended to increase propionate concentrations (*p* = 0.08). In addition, while the molar proportions of propionate were decreased by the addition of protease in the diet with adequate protein, adding protease to the low protein diet resulted in an increase in propionate (*p* = 0.02 for the interaction diet × protease). Branched chained fatty acids, such as isovalerate, isobutyrate, and valerate, are primarily attributed to protein fermentation [39]; however, none of the treatments impacted either valerate or isobutyrate. For isovalerate, a trend was observed for both the type of diet and for the inclusion of protease (*p* = 0.09). More specifically, excreta from birds fed the adequate protein diet had a greater proportion of isovalerate than from birds fed the low protein diet (0.73% vs. 0.50%, respectively); and isovalerate proportions for no protease versus 200 g/t protease were 0.74% and 0.49%, respectively. Assuming that protease increases protein digestibility, the amino acids and polypeptides absorbed by the chicken would increase. In this scenario, protease could decrease the amount of amino acids available to bacteria, potentially decreasing the total and individual short chain fatty acid concentrations [42,43]. However, in the present study, protease had essentially no effect on short chain fatty acid concentrations. The lack of impact on excreta ammonia and short chain fatty acid concentrations further contradicts our original hypothesis that protease would improve protein digestibility.

### 3.3. Microbial Diversity of Excreta Microbiota

**Figure 1:**
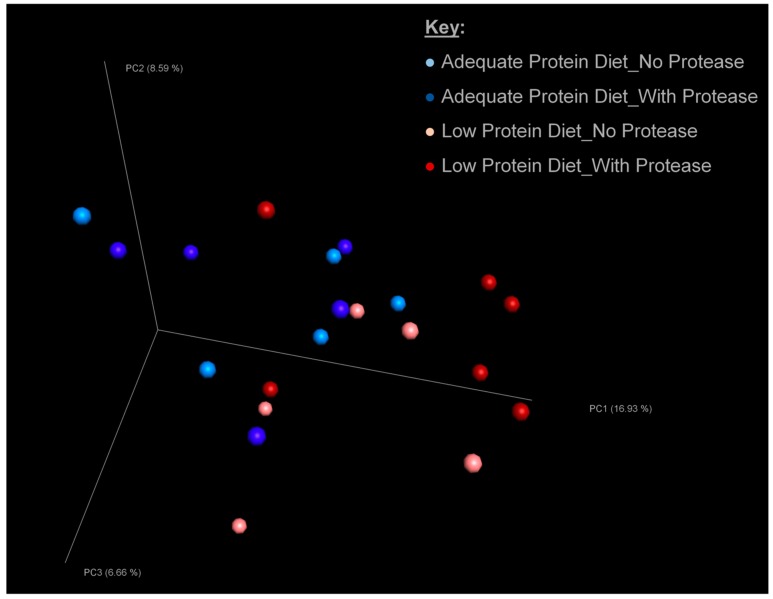
Principal coordinate analysis of β-diversity among sample groups. Bonferroni-corrected differences between treatments were not significant (*p* = 0.99).

Diet had little influence on the alpha diversity indices (Table 4). The two microbial richness indices shown—number of observed OTUs and Chao1—as well as Faith’s phylogenetic diversity index were not significantly changed by diet. However, another microbial diversity index (Shannon diversity index) tended to be lower (*p* = 0.07) in the excreta of chicks fed the low protein diet. Additionally, the microbial population tended to be more evenly distributed in the adequate protein diet compared with the low protein diet (Evenness *p*-value = 0.06). Inclusion of protease had some effects on both richness and diversity of the microbial populations: the number of observed OTUs was increased (*p* = 0.04), and Chao1 tended to be increased (*p* = 0.09) when protease was added to the diet. Similarly, Faith’s phylogenetic diversity was increased (*p* = 0.05) by protease inclusion. No interactions between diet and protease were observed for any of the alpha diversity indices. The principal coordinate analysis describing β-diversity (Figure 1) showed no differentiation (*p* = 0.99) between the microbial populations of chickens fed adequate protein diets and those fed low protein diets. Likewise, the inclusion of protease resulted in no β-diversity changes (*p* = 0.99). The first three principal components accounted for 32.18% of the variance.

### 3.4. Microbial Composition of Excreta Microbiota

As shown in Table 5, *Firmicutes* (40.9–53.6%) had the highest relative abundance, followed by *Proteobacteria* (34.0–53.9%), *Bacteroidetes* (2.8–10.8%), and *Actinobacteria* (0.3–1.1%). Tong et al. [44] and Singh et al. [45] reported that chickens at age 6-7 weeks had the following bacterial relative abundance in their excreta: *Proteobacteria* (46.4–78.8%), *Firmicutes* (12.0–27.5%), *Bacteroidetes* (7.1–27.2%), and *Actinobacteria* (0.8–1.9%) [46,47]. Oakley and Kogurt found that *Firmicutes* dominated the microbiota almost exclusively after week 1, and their study ended after 6 weeks [11]. In studies involving pasture-raised chickens, Lourenco et al. also found a predominance of *Firmicutes* in both the cecal contents and excreta of broilers, even at earlier ages (i.e., one-day-old chicks), regardless of their diets [46,47]. Therefore, it appears that the age of the bird, the surrounding environment, diet, and genetics all impact the composition of the chicken microbiota and account for differences in their microbial populations [10,16,48].

Diet tended to decrease the abundance of Actinobacteria (*p* = 0.09), while protease tended to decrease abundance of *Proteobacteria* (*p* = 0.09) and increase the presence of *Bacteroides* (*p* = 0.09); however, neither diet nor protease significantly changed *Firmicutes* (*p* ≥ 0.29). Excreta *Firmicutes* and *Bacteroides* have been linked to nutrient absorption. An increase in *Firmicutes* could lead to greater nutrient absorption, whereas an increase in *Bacteroides* could decrease nutrient absorption [11,49]. The ratio of *Firmicutes* to *Bacteroides* in our study had high variability both within and between treatment groups (SEM = 69.56); therefore, no diet or protease effect was observed. Protease could have decreased nutrient absorption, as indicated by the increased presence of *Bacteroides*; however, since protease had no effect on overall body weight gain and FCR, any changes that might have occurred in nutrient absorption were likely of small magnitude.

At the genus level, an unclassified genus from the family *Enterobacteriaceae* accounted for most of the relative abundance (27.1–45.3%) in the excreta of the chicks, followed by *Lactobacillus* (11.6–25.8%), *Enterococcus* (6.8–12.6%), *Bacteroides* (1.2–10.2%), an unclassified member of the family *Plancoccaceae* (1.6–7.3%), *Klebsiella* (2.5–5.6%), *Ruminococcus* (2.3–4.8%), *Proteus* (0.6–4.8%), *Acinetobacter* (1.0–3.7%), and other minor genera (Table 6). Bacterial substrate preferences, growth requirements, and nutrient availability in the digesta determined this distribution of the bacterial population within the chick’s microbiota [12].

An unidentified member of the family of *Enterobacteriaceae* accounted for the majority of bacteria in this study. The classification of *Enterobacteriaceae* includes 44 genera and 107 species. Genera *Alterococcus, Brenneria, Buttiauxella, Cedecea, Citrobacter, Edwardsiella, Erwinia, Escherichia, Leminorella, Pantoea, Pectobacterium, Photorhadus, Salmonella, Serratia, Shigella, Xenorhabdu,* and *Yersisna* are some of the best-known members of the *Enterobacteriaceae* family. All require glucose, vitamins and amino acids for growth [50]; however, the unclassified member of the family *Enterobacteriaceae* detected in the present study was not significantly influenced by diet (*p* = 0.41), protease (*p* = 0.21), or their interaction (*p* = 0.14).

*Lactobacillus* populations were not affected by protease or diet, but an interaction between diet and protease was observed (*p* = 0.02): while the abundance of *Lactobacillus* was decreased by the presence of protease in the adequate protein diet, the opposite effect was observed in the low protein diet. *Lactobacillus* species are found throughout the digestive tract, predominantly in the small intestine. They are thought to contribute to nutrient absorption and are involved with bile salt hydrolysis [16,51]. *Lactobacillus* are gram-positive and facultatively anaerobic, and are fastidious with complex nutritional requirements, including fermentable carbohydrates, amino acids, peptides, vitamins, salts, and fatty acids; however, each *Lactobacillus* species usually has characteristic nutrient requirements, and require a different profile of amino acids [52]. Apajalahti and Vienola hypothesized that protease would decrease lactobacilli located in the small intestine [42]; however, our data do not support this hypothesis, as protease did not consistently decrease excreta lactobacilli.

The reduction in protein in the diet decreased the relative abundances of *Proteus* and *Acinetobacter* (*p* = 0.01), which is logical given that both *Proteus* and *Acinetobacter* utilize amino acids as substrates. *Proteus* are gram-negative, facultatively anaerobic bacteria, known to deaminate phenylalanine and tryptophan, decompose tyrosine, hydrolyze urea, and catabolize glucose and other carbohydrates [53]. *Acinetobacter* bacteria are gram-negative and aerobic. Most *Acinetobacter* grow in media containing a single source of carbon and energy, and they frequently use amino acids as their sole source of nitrogen [54].

### 3.5. Microbial Correlation with Feed Efficiency

**Figure 2:**
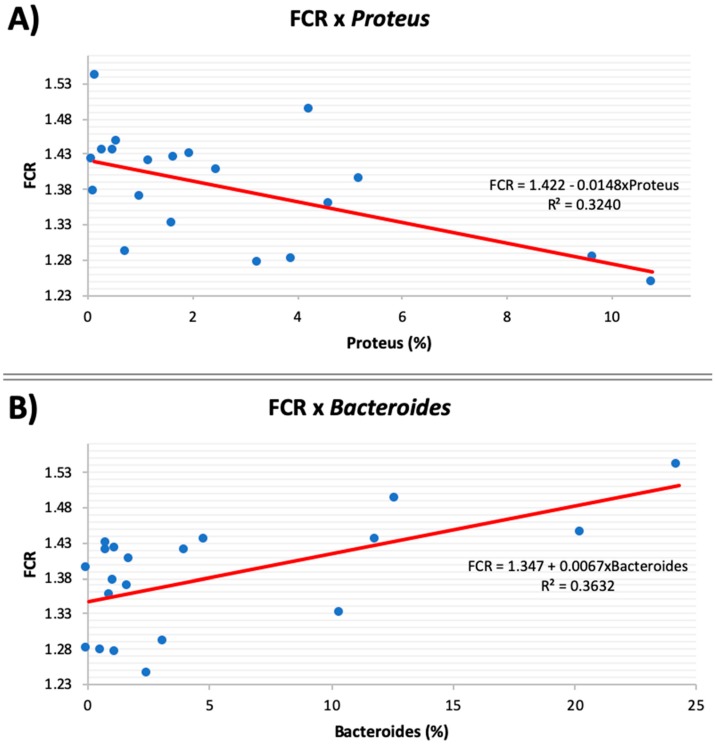
Relationship between overall feed conversion ratio (FCR) and abundance of the genera *Proteus* (**A**) and *Bacteroides* (**B**). *Proteus* had a negative correlation (ρ = −0.57; *p* = 0.009) with FCR. *Bacteroides* had a positive correlation (ρ = 0.60; *p* = 0.005) with FCR.

Regression analysis identified strong associations between the genera *Proteus* and *Bacteroides* with overall FCR, and Figure 2 summarizes those relationships. Our data revealed that while *Bacteroides* had a positive relationship with FCR (ρ = 0.60; *p* = 0.005), *Proteus* had a negative relationship (ρ = −0.57; *p* = 0.009). Since in the present study FCR was expressed as the ratio of feed consumed:body weight gain, lower values indicate a better FCR (more efficient birds). Consequently, a greater abundance of *Bacteroides* was associated with poorer feed efficiency, whereas a greater abundance of *Proteus* was associated with improved feed efficiency. Our results are in line with the ones reported by Singh et al. [45], who found a greater abundance of *Bacteroides* in the excreta of broilers that had poorer FCR. In humans, Jumpertz et al. [49] found that an increase in the population of *Bacteroides* led to a decrease in nutrient absorption. Regarding the genus *Proteus*, Singh et al. [55] reported that birds with better feed conversion had a lower abundance of this genus in their excreta; however, in their study, FCR was assessed during the last two weeks of broilers’ life cycle (from 35 to 49 days-old). In contrast, in our study, FCR was assessed in the first two weeks of boilers’ life, which may explain these contradictory results. Furthermore, the association of *Proteus* with improved animal performance has been demonstrated: the addition of *Proteus* spp. to the diets of fish at 4 g/kg resulted in improved weight gain, increased body length, and improved FCR [56], which is in line with our results.

## 4. Conclusions

Chicks fed a diet marginally deficient in amino acids had a reduced growth rate and poorer feed efficiency. Supplemental protease addition to this amino acid-deficient diet did not improve growth performance as expected. Overall, there was no effect of protease addition in either the adequate or low protein diets. Furthermore, protease demonstrated no effect on excreta concentration of ammonia and short chain fatty acids, suggesting that it had no effect on protein degradation in the gastrointestinal tract. However, the addition of protease induced some changes in microbial richness and diversity, and tended to alter some specific microbial taxa (e.g., increase the abundance of *Bacteroidetes*). Unlike protease, the level of protein in the chicks’ diet had a significant impact on their growth performance, as final body weight, ADG, and FCR were all significantly improved in the diet with adequate levels of protein. Moreover, despite the lack of substantial effects on excretal ammonia, short chain fatty acids, and on most alpha diversity metrics, increasing the level of protein in the diet resulted in increased abundances of *Acinetobacter* and *Proteus*, and this latter genus of bacteria was found to be significantly correlated with improved feed efficiency in the young chicks.

## Figures and Tables

**Table 1:** Composition and nutrient contents of the diets offered to chicks.

Item	Diet ^1^	
Ingredient, % of Inclusion	Adequate Protein	Low Protein
Corn	53.85	63.25
Soybean meal	29.70	21.75
Dried distillers’ grains with solubles	10.00	10.00
Fat	2.52	1.08
Limestone	1.29	1.31
Dicalcium phosphate	1.47	1.52
Salt	0.30	0.30
Vitamin premix ^2^	0.25	0.25
Mineral premix ^3^	0.075	0.075
L-lysine	0.27	0.28
DL-methionine	0.28	0.18
**Calculated Chemical Composition**		
Metabolizable Energy, kcal/kg	3010	3010
Crude Protein, %	22.30	19.23
Ether Extract, %	5.56	4.40
Crude Fiber, %	3.23	3.13
Ca, %	0.90	0.90
Available P, %	0.40	0.45
Lysine, %	1.30	1.10
Total sulfur amino acids, %	0.96	0.81
Threonine, %	0.86	0.73
Tryptophan, %	0.28	0.23
**Analyzed Composition** ^4^		
Crude Protein, %	20.83	18.95
Lysine, %	1.31	1.15
Methionine, %	0.55	0.46
Cysteine, %	0.34	0.30
Threonine, %	0.75	0.67
Tryptophan, %	0.23	0.21

^1^ Protease was added to both diets to create 2 additional treatments. ^2^ Supplied per kilogram of diet: vitamin A, 5511 IU; vitamin D3, 1102 ICU; Vitamin E, 11.02 IU; vitamin B12, 0.01 mg; Biotin, 0.11 mg; Menadione, 1.1 mg; Thiamine, 2.21 mg; Riboflavin, 4.41 mg; d-Pantothenic Acid, 11.02 mg; Vitamin B6, 2.21 mg; Niacin, 44.09 mg; Folic Acid, 0.55 mg; Choline, 191.36 mg. ^3^ Supplied per kilogram of diet: Mn, 107.2 mg; Zn, 85.6 mg; Mg, 21.44 mg; Fe, 21.04; Cu, 3.2 mg; I, 0.8 mg; Se, 0.32 mg. ^4^ Values represent the average of duplicate analysis conducted at the University of Missouri Experiment Station Chemical Laboratories.

**Table 2:** Effect of diet ^1^ and protease on chick performance from day 0 to 14.

Item	Adequate Protein		Low Protein		SEM ^3^	*p*-Value ^2^		
	No Protease	200 g/t Protease	No Protease	200 g/t Protease		Diet	Protease	Diet × Protease
Body weight day 0, g	48.5	48.2	48.9	49.0	0.33	*0.07*	0.91	0.50
Body weight day 7, g	181.7	178.4	171.8	174.5	3.24	**0.05**	0.94	0.37
Body weight day 14, g	416.1	423.1	392.8	383.4	12.92	**0.03**	0.93	0.53
ADG ^4^ day 0 to 7, g	19.0	18.6	17.6	17.9	0.45	**0.03**	0.95	0.39
ADG ^4^ day 7 to 14, g	33.5	35.0	31.6	29.8	1.60	**0.04**	0.93	0.33
ADG ^4^ day 0 to 14, g	26.3	26.8	24.6	23.9	0.92	**0.02**	0.93	0.52
Daily feed intake day 0 to 7, g	23.0	22.7	23.0	24.4	0.59	0.16	0.39	0.19
Daily feed intake day 7 to 14, g	46.0	49.7	46.7	44.4	2.35	0.34	0.78	0.21
Daily feed intake day 0 to 14, g	34.5	36.2	34.9	34.4	1.37	0.61	0.67	0.43
FCR ^5^ day 0 to 7	1.21	1.22	1.31	1.36	0.01	**<0.001**	**0.03**	0.19
FCR ^5^ day 7 to 14	1.37	1.42	1.48	1.51	0.04	**0.04**	0.41	0.80
FCR ^5^ day 0 to 14	1.31	1.35	1.42	1.45	0.03	**<0.01**	0.27	0.82

^1^ Adequate protein: diet with adequate levels of essential amino acids; low protein: diet with marginally deficient levels of essential amino acids. ^2^ *p* ≤ 0.05 are bolded to highlight significant differences. Trends, italicized, are defined as 0.10 ≥ *p* > 0.05. ^3^ SEM = standard error of the mean. ^4^ ADG = average daily body weight gain. ^5^ FCR = feed conversion ratio. Calculated as g of feed intake ÷ g of body weight gain.

**Table 3:** Effect of diet ^1^ and protease on the concentration of ammonia-N, total short chain fatty acids (SCFA), and molar proportions of SCFA (mol/100 mol) in the excreta of chicks.

Item	Adequate Protein		Low Protein		SEM ^3^	*p*-Value ^2^		
	No Protease	200 g/t Protease	No Protease	200 g/t Protease		Diet	Protease	Diet × Protease
Ammonia-N (mg/g)	1.17	1.06	0.88	1.05	0.10	0.13	0.76	0.17
Total SCFA, m*M*	58.27	58.70	46.52	52.05	5.11	*0.09*	0.57	0.63
Acetate	89.29	89.49	89.89	87.80	1.31	0.68	0.48	0.39
Propionate	1.31	0.97	1.16	1.99	0.23	*0.08*	0.31	**0.02**
Butyrate	6.88	8.04	6.22	8.14	1.14	0.81	0.20	0.74
Isobutyrate	0.43	0.27	0.22	0.29	0.06	0.14	0.50	0.09
Valerate	0.32	0.10	0.61	0.50	0.31	0.24	0.54	0.79
Isovalerate	0.95	0.52	0.53	0.47	0.13	*0.09*	*0.09*	0.18
Caproate	0.81	0.65	1.38	0.81	0.82	0.67	0.66	0.80

^1^ Adequate protein: diet with adequate levels of essential amino acids; low protein: diet with marginally deficient levels of essential amino acids. ^2^ *p* ≤ 0.05 are emboldened to highlight significant differences. Trends, italicized, are defined as 0.10 ≥ *p* > 0.05. ^3^ SEM = standard error of the mean.

**Table 4:** Effect of diet ^1^ and protease on α diversity indices at 97% similarity after rarefaction to 56,855 sequences per sample.

Item	Adequate Protein		Low Protein		SEM ^3^	*p*-Value ^2^		
	No Protease	200 g/t Protease	No Protease	200 g/t Protease		Diet	Protease	Diet × Protease
Observed OTUs	1132	1184	919	1206	76.76	0.23	**0.04**	0.15
Chao1	1992	2052	1600	2030	135.79	0.15	*0.09*	0.19
Phylogenetic diversity ^4^	47.2	48.9	39.5	49.7	2.83	0.24	**0.05**	0.15
Shannon index	5.0	5.1	4.3	4.9	0.23	*0.07*	0.15	0.23
Evenness	0.49	0.50	0.44	0.48	0.02	*0.06*	0.22	0.29

^1^ Adequate protein diet: diet with adequate levels of essential amino acids; low protein diet: diet with marginally deficient levels of essential amino acids. ^2^ *p* ≤ 0.05 are emboldened to highlight significant differences. Trends, italicized, are defined as 0.10 ≥ *p* > 0.05. ^3^ SEM = standard error of the mean. ^4^ Faith’s Phylogenetic Diversity Index.

**Table 5:** Effect of diet ^1^ and protease on the relative abundance of the main bacterial phyla ^2^.

Phyla	Adequate Protein		Low Protein		SEM ^4^	*p*-Value ^3^		
	No Protease	200 g/t Protease	No Protease	200 g/t Protease		Diet	Protease	Diet × Protease
*Firmicutes*	46.5	46.9	40.9	53.6	5.93	0.93	0.29	0.31
*Proteobacteria*	48.4	45.0	53.9	34.0	6.42	0.67	*0.09*	0.22
*Bacteroidetes*	2.8	6.5	4.0	10.8	2.93	0.36	*0.09*	0.61
*Actinobacteria*	1.1	0.5	0.1	0.3	0.32	0.09	0.55	0.20
*Firmicutes:Bacteroidetes*	58.7	140.5	25.4	18.9	69.56	0.28	0.60	0.54

^1^ Adequate protein: diet with adequate levels of essential amino acids; low protein: diet with marginally deficient levels of essential amino acids. ^2^ Phyla with overall relative abundance ≥ 0.10%. ^3^ *p* ≤ 0.05 are emboldened to highlight significant differences. Trends, italicized, are defined as 0.10 ≥ *p* > 0.05. ^4^ SEM = standard error of the mean.

**Table 6:** Effect of diet ^1^ and protease on relative abundance (%) of the main bacterial genera ^2^.

Genera	Adequate Protein		Low Protein		SEM ^4^	*p*-Value ^3^		
	No Protease	200 g/t Protease	No Protease	200 g/t Protease		Diet	Protease	Diet × Protease
Unclassified, Family *Enterobacteriaceae*	30.1	31.7	45.3	27.1	6.29	0.41	0.21	0.14
*Lactobacillus*	22.3	11.6	13.0	25.8	4.39	0.58	0.82	**0.02**
*Enterococcus*	8.1	12.6	7.1	6.8	2.03	0.12	0.32	0.26
*Bacteroides*	1.2	5.4	3.9	10.2	3.00	0.23	*0.10*	0.74
Unclassified, Family *Planococcaceae*	5.1	7.3	6.6	1.6	3.14	0.52	0.67	0.27
*Klebsiella*	5.3	3.9	5.6	2.5	1.29	0.66	*0.10*	0.52
*Ruminococcus*	2.6	2.3	3.6	4.8	1.43	0.23	0.75	0.62
*Proteus*	4.8	4.2	1.2	0.6	1.16	**0.01**	0.61	0.97
*Acinetobacter*	2.4	3.7	1.0	1.1	0.59	**0.01**	0.26	0.32
Unclassified, Family *Ruminococcaceae*	1.8	1.0	1.8	2.9	0.82	0.26	0.82	0.27
Unclassified, Family *Alcaligenaceae*	3.7	0.1	0.1	1.5	2.00	0.58	0.58	0.22
Unclassified, Order *Clostridiales*	0.5	1.0	1.7	2.1	0.80	0.17	0.53	0.93
*Blautia*	0.9	0.9	0.8	1.3	0.39	0.69	0.53	0.45
*Oscillospira*	0.2	0.4	0.8	1.6	0.58	0.15	0.40	0.62

^1^ Adequate protein: diet with adequate levels of essential amino acids; low protein: diet with marginally deficient levels of essential amino acids. ^2^ Genera with overall relative abundance ≥ 0.70%. Bacteria not identified at the genus level are presented at the subsequent taxonomic level. ^3^ *p* ≤ 0.05 are emboldened to highlight significant differences. Trends, italicized, are defined as 0.10 ≥ *p* > 0.05. ^4^ SEM = standard error of the mean.
